# Integrative Metabolomics to Identify Molecular Signatures of Responses to Vaccines and Infections

**DOI:** 10.3390/metabo10120492

**Published:** 2020-11-30

**Authors:** Joann Diray-Arce, Maria Giulia Conti, Boryana Petrova, Naama Kanarek, Asimenia Angelidou, Ofer Levy

**Affiliations:** 1 Precision Vaccines Program, Division of Infectious Diseases, Boston Children’s Hospital, Boston, MA 02115, USA; mariagiulia.conti@uniroma1.it (M.G.C.); asimenia.angelidou@childrens.harvard.edu (A.A.); 2 Department of Pediatrics, Harvard Medical School, Boston, MA 02115, USA; boryana.petrova@childrens.harvard.edu (B.P.); naama.kanarek@childrens.harvard.edu (N.K.); 3 Department of Maternal and Child Health, Sapienza University of Rome, 5, 00185 Rome, Italy; 4 Department of Pathology, Boston Children’s Hospital, Boston, MA 02115, USA; 5 Department of Neonatology, Beth Israel Deaconess Medical Center, Boston, MA 02115, USA; 6 Broad Institute of MIT & Harvard, Cambridge, MA 02142, USA

**Keywords:** metabolomics, vaccines, infections, integrative metabolomics, systems biology, diagnosis, response detection

## Abstract

Approaches to the identification of metabolites have progressed from early biochemical pathway evaluation to modern high-dimensional metabolomics, a powerful tool to identify and characterize biomarkers of health and disease. In addition to its relevance to classic metabolic diseases, metabolomics has been key to the emergence of immunometabolism, an important area of study, as leukocytes generate and are impacted by key metabolites important to innate and adaptive immunity. Herein, we discuss the metabolomic signatures and pathways perturbed by the activation of the human immune system during infection and vaccination. For example, infection induces changes in lipid (e.g., free fatty acids, sphingolipids, and lysophosphatidylcholines) and amino acid pathways (e.g., tryptophan, serine, and threonine), while vaccination can trigger changes in carbohydrate and bile acid pathways. Amino acid, carbohydrate, lipid, and nucleotide metabolism is relevant to immunity and is perturbed by both infections and vaccinations. Metabolomics holds substantial promise to provide fresh insight into the molecular mechanisms underlying the host immune response. Its integration with other systems biology platforms will enhance studies of human health and disease.

## 1. Introduction 

Metabolites are small molecules (50 to 1500 Daltons) produced by regulatory mechanisms and during cellular processes or acquired from exogenous sources, including diet and xenobiotics such as drugs [1]. The metabolic profile provides a snapshot of the complex interplay between genome, environment, and intermediary processes [2]. Metabolites play critical roles in biological pathways and serve as valuable bioindicators of cell physiology [3].

As early as 1955, biochemical research provided the first perspective of a comprehensive cellular metabolome comprised of ~20 metabolic pathways [4]. As mass spectrometry evolved in the following decades, it dramatically expanded the range and detail of mapping biochemical charts [2]. “Metabolomics” thus emerged as a new frontier in systems biology [5]. Though this technology is powerful and comprehensive, the exact set of metabolites it provides depends on the precise technique employed and its sensitivity and specificity. 

Within the framework of precision medicine and in comparison to other systems biology approaches such as transcriptomics (e.g., RNASeq) and proteomics, metabolomics provides a nearly instantaneous snapshot of metabolism. The metabolome rapidly responds to even minor stimulations, rendering metabolomics a powerful approach to assess quantitative responses to stress [6], nutritional changes [7], disease states [8], host–pathogen interactions [9], as well as short- and long-term metabolic effects of infection [10] and vaccination [11]. The multitude of metabolite changes across space and time and their impact on downstream biological processes produces a complex wealth of data that requires sophisticated detection methods, separation, and analyses based on molecular characteristics. Of note, there are still uncharted areas of the metabolome that may be key to the host response to infection [12,13,14,15,16] and vaccines [17], including the relatively underexamined lipid families assessed via “lipidomics,” a branch of metabolomics [18]. 

This review highlights metabolomics as an emerging tool for identifying signatures and pathways in the host response to infection and vaccination.

## 2. Metabolomics—An Emerging Tool to Complement Other Systems Immunology Platforms

Systems biology approaches that focus on a single class of molecules, such as transcripts (transcriptomics) or proteins (proteomics), can provide important but limited understanding of the biological mechanisms of disease [3]. Their combination with metabolomics provides insights into complementary and synergistic interactions at different cellular and molecular levels [19]. The integration of multi-omic variables addresses gaps in our current knowledge of disease pathogenesis and evolution, offers opportunities for early diagnosis, prevention, and potential treatment of disease, and allows gaining a holistic understanding of a dynamic biological system [20]. 

Recent systems immunology methodologies have enabled a comprehensive analysis of multiple immune system features in parallel, as well as the identification of cellular and molecular biomarkers not previously known to be relevant to immune responses [21]. Multi-omic integration of metabolomics with other systems biology platforms has enabled the comprehensive characterization of diseases and the identification of metabolic pathways involved in a range of pathologies, including cancer [22,23,24,25,26,27], chronic obstructive pulmonary disease [28], asthma [29,30], and sepsis [31,32]. The use of systems biology platforms together with metabolomics accelerates biomarker discovery and has been increasingly incorporated in preclinical study workflows such as those related to nutrition and diet [33,34,35,36,37,38]. Targeted metabolite assays, including those for eicosanoids, arginine, and citrulline, are currently performed for clinical use based on metabolites’ association with immune regulatory pathways [39,40,41] and metabolic diseases [42,43,44]. In addition, there are existing metabolite tests that are routinely available in the U.S. for point-of-care testing, including those for glucose, 1,5 anhydroglucitol, carbohydrates, lipids, and amino acid (AA) panels to assess glycemic disorders [45].

**Figure 1:**
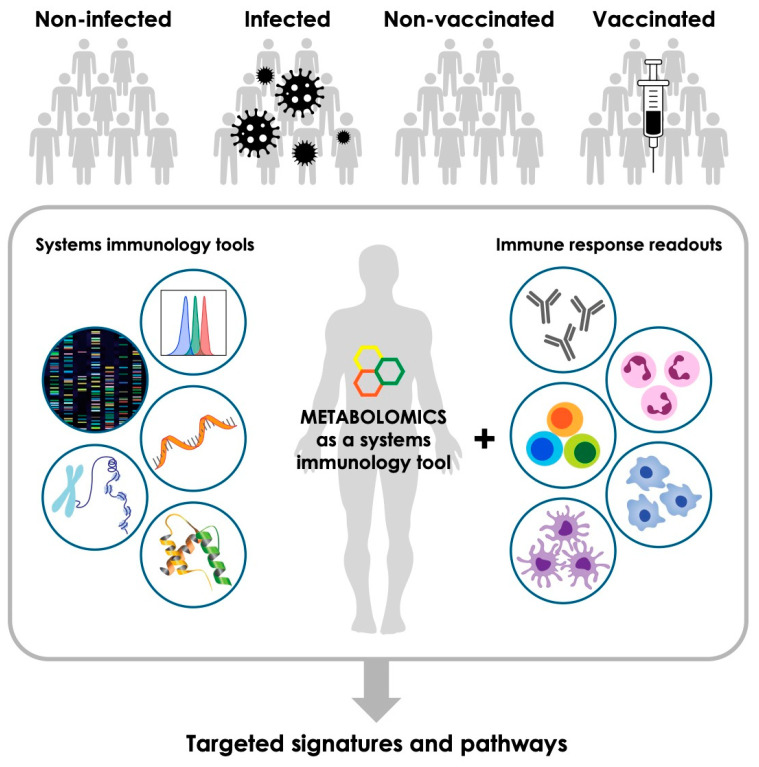
Metabolomics is an emerging tool that can complement other high-throughput systems immunology methods and immune response readouts to identify infection and vaccination biomarkers.

The potential contribution of metabolomics in the context of infectious diseases and host–pathogen interactions appears particularly promising. Host–pathogen interactions impact leukocyte immunometabolism, thereby shaping their response to infection. Plasm- or serum metabolomics can profile the metabolome in an infected patient, reflecting the systemic responses of diverse cell types in various organ systems affected by a specific pathogen and thereby identifying potential biomarkers of infection [46]. Finally, metabolomics can complement the characterization of complex systems measurements of the effect of a defined immune perturbation such as vaccination (Figure 1). Such an approach is particularly promising in the newborn, given our recent demonstration of marked changes in numerous metabolic pathways across the first week of human life [47], a time of marked susceptibility to infection and of receipt of multiple vaccines, such as via the Expanded Program on Immunization—which includes Bacille Calmette–Guérin vaccine, hepatitis B vaccine, and polio vaccine) [48,49]. In light of these proof-of-concept examples, further metabolomic discovery and targeted metabolite validation may provide novel biomarkers of infectious diseases and successful immunization. 

## 3. Immunometabolism

The interplay between metabolism and the immune response is increasingly recognized, and distinct metabolic needs and demands define responses to infection and vaccination [50]. Immunometabolism is a rapidly evolving field of immunology, and the metabolites produced by leukocytes serve as potent immune signaling molecules, during primary (innate), trained (innate memory), and classical adaptive immunity [51]. Trained immunity, the phenomenon of antigen-agnostic memory responses by leukocytes subjected to various environmental threats, is an evolutionary process mediated by epigenetic modulations [52]. Global or targeted metabolomic analysis of pre-defined immune cell populations can lead to novel discoveries of metabolic pathways or molecules that serve as epigenetic or transcriptional regulators. For example, mammalian target of rapamycin (mTOR) signaling, primarily by activating the transcription factor hypoxia-inducible factor-1 (HIF-1α), increases aerobic glycolysis and regulates the differentiation of CD4^+^ T cells, favoring their differentiation into Th17 rather than Treg cells, and the production of pro-inflammatory cytokines in response to T cell receptor activation [53].

In contrast, adenosine monophosphate-activated protein kinase (AMPK), an energy sensor kinase, serves as an immunometabolic checkpoint in T cell development and effector responses as well as in memory T cell differentiation by regulating the metabolic switch from aerobic glycolysis to oxidative phosphorylation of lipids [54]. As is the case for all the body’s cells, leukocytes use lipids to synthesize cell membranes and for posttranslational modifications of proteins; therefore, lipid metabolism is relevant to immune responses [55], including those related to epigenetic reprogramming [56]. Integration of metabolomics with other systems biology platforms can enrich the discovery, characterization, and validation of immune signatures and networks [47]. Moreover, characterizing the impact of infection and immunization on the immunometabolism of leukocyte populations may inform the discovery and development of target-based therapeutics and vaccines.

## 4. Impact of Infection on Host Metabolic Signatures

During infection, several metabolic changes occur, with reciprocal effects between the pathogen and the host. Metabolic adaptations occurring in eukaryotic hosts upon acute infection by bacterial or viral pathogens are complex, as the pathogen competes for host nutrients and other metabolites to satisfy its bioenergetic and biosynthetic requirements. In contrast, the host response is aimed at the elimination of the invading pathogen [57]. Most microbes enhance specific anabolic pathways in the host and are highly dependent on these alterations, such that the characterization of these metabolic alterations may inform diagnostic, prognostic, and therapeutic applications [58]. Select studies investigating metabolic signatures of infection in the human host are listed in Table 1.

Common metabolic reactions are essential to host cell defense [93] to prevent microbial access to nutrients [94]. Generic common responses triggered by intracellular bacterial pathogens in host innate immune cells include: (a) induction of host cell reactive oxygen species (ROS) and reactive nitrogen intermediates (RNI) [95,96], (b) enhancement of glucose uptake, which stimulates host cells’ anabolic activity [58,97,98], (c) a switch to enhanced glutaminolysis and the citrate lyase reaction [99,100], enhancing fatty acid/lipid biosynthesis, and (d) an overall increase in lipid metabolism, especially the biosynthesis of steroids and eicosanoids [101].

*Mycobacterium tuberculosis* (Mtb), the causative agent of tuberculosis (TB), is an example of the metabolic adaptation of a bacterium to the host environment, as this pathogen has learned to survive in the host causing a latent infection (LTB) which can progress to active TB disease in ~10% of latently infected individuals. Carbon metabolism is a major determinant of the pathogenicity of Mtb, as demonstrated in animal models, where the lack of carbon sources causes failed replication and survival. It is essential to fuel Mtb growth [102]. Untargeted metabolite profiling of Mtb growing on ^13^C-labeled carbon substrates revealed that Mtb could simultaneously catabolize multiple carbon sources (e.g., dextrose, acetate, and glycerol) to augment its growth [64]. The sphingolipid metabolic pathway is another established mediator of the host response to TB [75]. Sphingolipids are fundamental building blocks for cell membranes, are important to immune signaling, and are major constituents of the mucus secreted by lung alveolar epithelial cells [13]. Metabolic signature identification in pulmonary active TB, employing high-resolution plasma metabolomics (HRM), revealed that tryptophan metabolism is highly regulated during TB infection and disease and is characterized by increased catabolism to kynurenine, which occurs in both latent and active TB patients [63]. Increased tryptophan catabolism may enable the survival of Mtb at the site of infection by modulating CD4^+^ T cell responses, inducing immune tolerance and bacterial persistence, and could also protect the host from excessive inflammation. Metabolomics can identify immunometabolic pathways associated with TB progression, discriminating active TB from latent TB [71]. Fatty acid metabolic networks are critical in TB progression. Mtb favors fatty acids as a cellular nutrient source and the expression of multiple genes dedicated to fatty acid metabolism, at higher levels than those induced by any other microorganism [72,73]. Of note, higher serum concentrations of glutamate, sulfoxy-methionine, and aspartate and lower serum levels of glutamine, methionine, and asparagine are noted in active TB patients compared to latent TB subjects or healthy controls [65]. Lastly, the amino-acyl tRNA pathway is associated with the progression of TB infection to disease, with progressors demonstrating a significant decrease of AA levels compared to controls [71].

Viruses depend on the host cell to obtain macromolecules and on host biosynthesis machinery to replicate; they utilize host cell metabolism according to their specific needs [103]. Viral reprogramming of host metabolism contributes to viral pathogenesis by fueling viral proliferation and survival, enhancing access to free AA, fatty acids, and host-derived lipid membranes, eventually augmenting intercellular signaling promoting evasion of the host’s immune system [104]. Some of the significant cellular metabolic pathways, including glycolysis, fatty acid synthesis, and glutaminolysis, are significantly altered by multiple virus families such as HCMV, HCV, HSV-1 poliovirus [103]. Serum metabolomics of chikungunya and/or dengue (co)infection revealed that glycine, serine, threonine, galactose, and pyrimidine metabolism are the most perturbed host pathways in both single and co-infection conditions [76]. Tryptophan metabolites serotonin and kynurenine are differently enriched in patients with dengue hemorrhagic fever (DHF) and their presence may be used in combination with the levels of interferon (IFN)-γ for early prognostication of DHF [78]. Analysis of influenza A virus (IAV)-infected cells revealed alterations in several metabolites of the purine, lipid, and glutathione pathways, resulting in the acceleration of viral replication [16].

Retroviruses directly alter host cell metabolism as well. Metabolites involved in glycolysis were increased in human immunodeficiency virus (HIV)-infected CD4^+^ T cells [81] but decreased in infected macrophages [82]. Macrophages generally maintain long-term infection, while CD4^+^ T cells most often are effectors of the acute lytic infection, which may explain the differences. HIV metabonomic studies utilizing biofluids from HIV-infected patients and controls have been used to identify HIV infection biosignatures, disease progression, and immunological responses to treatment [105]. Plasma metabolomics demonstrated deficient concentrations of sphingomyelins and dopamine, in parallel with high levels of dicarboxylicacylcarnitines, L-aspartate, and many plasmalogen/plasminogen phosphatidylcholines in the blood of HIV-1-infected individuals compared with controls [83]. Of note, patients defined as immunological non-responders, demonstrated a downregulation of β-oxidation, important in T cell survival, and sphingosine-1-phosphate-phosphatase-1 activity, which is involved in lymphocyte egress from lymphoid organs and the bone marrow. In contrast, acyl–alkyl-containing phosphatidylcholines and alkylglyceronephosphate synthase levels were elevated [83], suggesting that metabolomics can predict a potential rapid disease progression or inadequate antiretroviral immunological responses. 

Interestingly, the interaction between glucose metabolism and the inflammatory cytokine network might trigger the host’s systemic inflammatory response. Metabolomics of peripheral blood mononuclear cells (PBMCs) challenged with IAV or H1N1 demonstrated that an increase in glucose metabolism promotes viral replication and cytokine production [86]. Plasma metabolomics of study participants with H1N1 influenza A pneumonia or bacterial community-acquired pneumonia (CAP) demonstrated metabolic changes linked to H1N1 pneumonia compared with CAP, including decreased in citrate, fumarate, alanine, and tyrosine levels and increased carnitine, glycine, and acetoacetate levels [85]. 

In the context of the severe acute respiratory syndrome coronavirus-2 (SARS-CoV-2) pandemic, advances in metabolomics can shed light on the pathogenesis of coronavirus disease 2019 (COVID-19) that could inform the development of novel therapeutics. Patients with COVID-19 exhibit changes in serum tryptophan metabolism and increased circulating levels of glucose and free fatty acids (FFA), consistent with altered carbon homeostasis, compared with SARS-CoV-2-negative controls. Interestingly, these findings correlate with the detection of clinical laboratory markers of inflammation (interleukin-6 (IL-6) and C-reactive protein) and renal function (i.e., blood urea nitrogen) [89]. Plasma lipid alterations (i.e., enhanced levels of sphingomyelins (SMs) and monosialodihexosylgangliosides (GM3s) and reduced diacylglycerols (DAGs) associated with COVID-19, detected by targeted and untargeted tandem mass spectrometry analysis of the plasma lipidome and metabolome in mild, moderate, and severe COVID-19 patients and unaffected controls, suggest that SARS-CoV-2 might take advantage of host-derived lipid membranes [90], as it has been described for other coronaviruses [91]. Metabolic signatures may allow the early detection of infected patients at risk for severe disease before the appearance of severe clinical manifestations. Proteomic and metabolomic profiling of sera from COVID-19 and control individuals demonstrated that the elevation of glucose, glucuronate, bilirubin degradation products, and four bile acid derivatives potentially indicates compromised liver detoxification function in patients with severe COVID-19 disease [14]. Plasma metabolite and lipid alterations are more extensive in fatal COVID-19 cases than in patients with severe and mild symptoms [92]. Compared to healthy volunteers, the carbohydrate pathway metabolites malic acid and glycerol 3-phosphate are diminished in symptomatic patients and demonstrated the most significant reduction in patients who died. Plasma lipidomic alterations relate to clinical symptoms of COVID-19: diglyceride (DG), FFA, and triglyceride (TG) concentrations increase with disease deterioration, while concentrations of phosphatidylcholines (PCs) decrease in patients with fatal COVID-19 [92].

## 5. Metabolic Signatures of Vaccine-Induced Responses

Systems vaccinology provides fresh insights into distinct age- and sex-specific vaccine-induced responses [106,107,108,109]. The application of metabolomics to vaccinology may identify metabolites that correlate with immunogenicity and inform the tailoring of vaccine regimens for distinct vulnerable populations [109]. Plasma metabolic components, including small molecules and lipids, have immunomodulatory effects that may impact vaccine immunogenicity and infection responses.

Systems vaccinology studies have uncovered metabolic pathways that correlate with vaccine immunogenicity and may have critical roles in immune response mechanisms [11,110,111] (Table 2). Metabolomic studies of influenza immunization of antibiotic-treated study participants demonstrated significant cofactor-/vitamin-related metabolism changes at Day 7 postvaccination compared to baseline [110]. Similarly, dysbiosis associated with bile acid metabolism occurred with antibiotic administration after influenza vaccines, and the IgG1 response was related to metabolic clusters of fatty acid metabolism. These observations suggest that perturbations of the microbiome may impact the levels of critical metabolites, thereby altering the immune response to vaccination [110]. 

A plasma metabolomic analysis of immune responses to herpes zoster (shingles) Zostavax vaccine demonstrated a strong association of transcriptomic pathways with multiple metabolic pathways, including lipid (e.g., glycerophospholipid, glycosphingolipid, and linoleate metabolism) and AA pathways (methionine and cysteine) at Day 3 postvaccination [11]. These metabolic pathways are also strongly associated with genes related to the MHC–TLR7–TLR8 cluster, antigen presentation, dendritic cell (DC) activation, and B cell signatures [11]. Purine and lysine metabolism were also found to overlap with transcriptomics data at Day 1 versus Day 0. While there was no association between transcriptomics and metabolomics at Day 7 postvaccination, the kinetics of vaccine-induced metabolic shifts may be dependent on gene expression [11]. 

In a phase III multicenter clinical trial, the Hantavax vaccination (Hantavirus vaccine) demonstrated dose-dependent upregulation of folate biosynthesis, nicotinate, nicotinamide, and arachidonic acid metabolism, thiamine levels, and pyrimidine metabolism in the high responder vaccine group [111]. In light of the known roles of metabolic pathways in immunity, Hantavax-induced perturbation of metabolites was thought to be related to immune function, including (a) folate biosynthesis with activation of cell-mediated immunity [112], (b) arachidonic acid metabolism with immune regulation [113], (c) thiamine elevation with enrichment of T cell differentiation and phagocytosis pathways [114], and (d) pyrimidine metabolism with cell-mediated immunity, T cell activation, and ultimately defeat of the microbial infection [115]. Metabolomics also identified elevation of key metabolites correlating with antibody responses, such as arginine, phenylalanine, cholesteryl nitrolinoleate, and octanoylcarnitine, in the high responder group. Elevated cholesteryl nitrolinoleate in high responders may reflect increased expression of inducible nitric oxide synthase (iNOS), as macrophage activation (e.g., by lipopolysaccharide and INF-γ) can increase the production of this metabolite [116]. Arginine and phenylalanine enhance macrophage activation for enhanced synthesis of nitric oxide (NO) and are key to the antiviral response against herpes simplex virus [117]. Such systems vaccinology studies have identified metabolic pathways that may be important to vaccine immunogenicity and may ultimately inform future vaccine design. 

Metabolomics is also being applied to study vaccine safety. A human plasma metabolomics study of 200 individuals pre- and post-small pox immunization discovered novel biomarkers for an adverse event following immunization (AEFI), including redness at the local site of vaccination or a severe allergic reaction [121]. Individuals who experienced clinically verified myocarditis or asymptomatic elevation of troponins were metabolically distinct from controls or those who only experienced systemic symptoms. Creatinine, fructose, phenylalanine, histidine, and serine metabolites were decreased in those with an identified adverse event, suggesting that certain vaccine adverse events may be metabolically mediated [121]. Metabolomics, coupled with machine learning approaches, may predict vaccines’ effects and potentially help avoid serious adverse events following immunization, thereby improving the risk/benefit ratio of immunization. In the race for a SARS-CoV-2 vaccine, metabolomic approaches may help identify vaccine candidates that demonstrate relatively low reactogenicity and superior protection. Leveraging systems biology approaches, including metabolomics, may further enhance the benefit-to-risk ratios and the quality of immunization programs, a topic of critical importance in an era of growing vaccine hesitancy. 

## 6. Shared Metabolic Pathways Across Infection and Vaccination Studies

Common metabolic pathways induced by infection and vaccination include AA (asparagine, glycine, methionine, threonine, tryptophan, glutamate, glutamine, and serine), carbohydrate (tricarboxylic acid (TCA) cycle, glycolysis, glucose, propanoate), nucleotide (purine), and lipid metabolism (bile acids). Remarkably, these are metabolic pathways known to shape the immune cell response [56,122]. 

Numerous reviews indicated that AA metabolism shapes the host’s physiology, growth, reproduction, and immunity. AA metabolites also serve as the energy source for cells and as regulators for cell signaling and host metabolism [123,124]. AA metabolites arginine, asparagine, and tryptophan are identified as central points of competition between the host and the pathogen [125]. Glutamine mediates the activation of Th1 and Th17 cells [126,127] and has also been implicated as an immunomodulatory nutrient that may represent a therapeutic target during severe infection [128]. Serine is required for optimal T cell expansion to support T cell activation and effector functions and supports de novo purine biosynthesis in proliferating T cells [101,129].

Immunometabolism involving carbohydrate pathways proposes that innate or adaptive naïve immune cells mainly rely on shifting oxidative phosphorylation to glycolysis (Warburg metabolism) upon immune activation [98]. Carbohydrate metabolism pathways are essential host factors influencing responses to bacterial and viral infections [130]. TCA cycle regulation demonstrates a mechanism of host defense against intracellular pathogens through hypoxia and impairment of STAT3 to reduce microbial replication [131]. These carbohydrate metabolic pathways have been extensively shown to promote innate immune cell survival, growth, and physiology [51], provide antimicrobial defense, and prevent hyperinflammatory responses [132,133]. Understanding this pathway during host–pathogen interaction is crucial, as each pathogen induces a specific metabolic program, suggesting that research directed at developing species-specific strategies to counter these infections will be fruitful [98]. 

Purine metabolites provide the necessary energy and cofactors to promote cell survival and proliferation [134]. Adenosine suppresses the proinflammatory response and promotes an anti-inflammatory response through purinergic receptors [135]. Deficiency or mutation of adenosine deaminase activity, a key enzyme for purine metabolite degradation, leads to the accumulation of adenosine, which increases the susceptibility to infection and autoimmunity [136,137]. Bile acids that primarily function in lipid metabolism play key roles in controlling the microbiota composition in the gut [138]. An altered bile acid pool has been associated with infection, suggesting a mechanistic link between the gut microbiota and metabolic syndromes. The interplay between host and pathogen in the gut microbial community underscores the critical involvement of this pathway, as described in vaccination [11,110,139] and infection studies [138,140].

## 7. Integrative Metabolomics—Challenges and Emerging Horizons 

The application of metabolomics for precision medicine is dependent on our ability to categorize infectious disease states or vaccine immunogenicity based on reproducible metabolic signatures that reflect specific, discernible phenotypes and can be used as a consistent readout [141]. For all its many strengths, metabolomics is lagging behind other systems biology fields concerning the standardization of protocols [44] and the routine usage of repositories for mass spectrometry data [142]. Close coordination between researchers, clinicians, and biomedical centers will be necessary to identify reproducible and generalizable metabolic signatures and realize the potential of metabolomics. 

Potential obstacles to the broader application of metabolomics for precision medicine have been the prohibitive cost of clinical metabolite profiling and the time-consuming nature of extraction and derivatization of certain metabolites. The typical cost of metabolomics at this time is hundreds of dollars per sample, such that the broad application of global metabolomics is unaffordable, limiting metabolomics-based applications to well-funded clinical trials and perhaps the most privileged patients. To overcome this limitation, technologies such as NMR, which is more affordable than mass spectrometry [143], could be employed, acknowledging the limitations in their detection abilities. Alternatively, detecting a single metabolite or a small group of well-characterized metabolites that were found to be informative of a patient’s clinical state can be performed using less costly techniques such as conventional HPLC. Fortunately, current efforts to reduce the cost of mass spectrometry-based methods by several companies might significantly reduce the metabolite profiling costs in the coming years. 

At present, the US Food and Drug Administration (FDA) regulations allow the application of metabolomics as a diagnostic tool for in vivo newborn screening of metabolic diseases [144] and for the in vitro identification of bacteria and fungi in clinical samples [145]. Although the FDA has recognized the potential of metabolomics as a promising technology for diagnostics as far back as 2008 [146] and acknowledged its importance and broad applicability to research and development, it has not approved the clinical application of metabolomics for precision medicine. Specific assays of diagnostic markers will have to be individually approved to ensure clinical readouts’ safety and accuracy. 

As the metabolomics research community finds ways to overcome the current limitations of this powerful technology, the way for personalized metabolic phenotyping will be paved [147,148], which may enable early diagnosis [149], informed treatment choices [150], and accurate prediction of response to therapy [151]. Of note, metabolomics data have advantages compared to data from other screening methods, as they directly measure drug metabolism, inflammation markers, and other ever-changing indicators of a patient’s disease status. Such metabolomics data are most useful when integrated with high-quality clinical data (such as ethnicity, age, sex, clinical history, etc.) and molecular data (genomic, transcriptomic, and proteomic data) to inform a comprehensive report of a patient’s health status.

Finally, robust structures and incentives for data sharing will be critical to the metabolomics field’s success. Extensive studies by investigators in the areas of infectious diseases, vaccinology, and metabolomics, coupled with data sharing, such as that fostered by the Human Immunology Project Consortium ImmPort database [152,153] (www.immport.org), will enable the discovery and validation of cellular and molecular signatures of infection and immunization. Eventually, when integrated with clinical information and validated in clinical contexts, such knowledge may help leveraging metabolic signatures to enhance the diagnosis and treatment of infectious diseases and inform vaccine discovery and development. Given the power of metabolomics and the growing evidence that metabolic pathways are relevant to immune responses, we predict that collaborative data verification and integration efforts will eventually enable the use of metabolic signatures to inform clinical decision-making. 

## 8. Conclusions

As the field of metabolomics evolves in accessibility, usability, and sophistication, metabolomic data will increasingly be integrated with clinical and other systems biology data to gain deep insights into the biology of health and disease. On addition to presenting metabolic pathways triggered by infections and vaccinations, our review also highlights metabolomics as a useful tool for discovering and identifying biomarkers of disease subtype and stage and treatment response. These biomarkers can be used to distinguish patients infected with different pathogens, predict vaccine-induced protection against these infections, and provide insight into human immunity. Given the emerging evidence that metabolism plays key roles in infection and vaccine responses, a growing number of metabolites will likely emerge as targets for developing biomarkers, predictive algorithms, therapeutic targets, and preventative modalities.

## Figures and Tables

**Table 1:** Metabolomics identifies biomarkers of human infections. Metabolites noted in more than one infection are in bold. HIV, human immunodeficiency virus, SARS-CoV-2, severe acute respiratory syndrome coronavirus 2, Covid19, coronavirus disease 2019, CSF, cerebrospinal fluid.

Pathogen Type Target	Pathogen (Infection)	Biosample Type	Technique Used	Examples of Metabolites or Metabolic Pathways Perturbed	References
**Bacteria**	*Clostridioides difficile* (infection)	stool	LC–MS, GC–MS	2-hydroxy-4-methypentanoic acid, 2TMS derivative, 4-methylpentanoic acid, allo-isoleucine, **bile acids**, chenodeoxycholic acid, cholenic acids, choleonoic acid, eicosatrienoic acid, **fatty acids**, fructose, glyceryl glycoside, isoleucine, **lysophosphatidylcholine** (16:0), phenylalanine, propylene glycol, ribitol, **sphingolipid**, **sphingomyelin**, tyrosine, tyrosol	[59,60]
**Bacteria**	*Escherichia coli* (associated urinary tract infection)	urine	H-NMR, LC–MS	acetate, amines, aspartic acid, cadaverine, citrate, glutamic acid, glycine, hippurate, trimethylamine, trimethylamine n-oxide	[61,62]
**Bacteria**	*Mycobacterium tuberculosis* (tuberculosis)	plasma, serum	LC–MS, FIA–MS, GC–MS	amino-acyl tRNA, asparagine, aspartate, citrulline, cysteine, gamma-glutamylglutamine, **fatty acid** metabolism, glutamate, glutamine, histidine, inosine, **kynurenine**, **lysophosphatidylcholines**, medium chain fatty acid, lysosome pathway, mannose methionine, protein digestion pathway, **sphingolipid**, **sphingosine-1-phosphate**, sulfoxymethionine, **tryptophan**, **urea**	[13,63,64,65,66,67,68,69,70,71,72,73,74,75]
**Virus**	Alphavirus (Chikungunya)	serum	H-NMR	2-hydroxycaproic acid, azelaic acid, carnitine, d-maltose, ethanol, galactitol, **galactose** metabolism and **citrate** cycle, gluconolactone, **glycine**, mandelic acid, methylguanidine, **serine**, **threonine** metabolism	[76]
**Virus**	Flavivirus (Dengue)	serum	GC–MS, LC–MS	acylcarnitines, **amino acids**, **bile acids**, chenodeoxyglycocholic acid, **galactose** and pyrimidine, **glycine**, glyoxylate and dicarboxylate, **kynurenine**, pentose phosphate pathway, phospholipids, propanoate, purines, **serine**, serotonin, starch and sucrose, **threonine**, uric acid	[76,77,78,79]
**Virus**	Lentivirus (human immunodeficiency virus/HIV)	CSF, CD4^+^ T cells, plasma	H-NMR, LC–MS, targeted LC–MS	acetate, citrate, **creatine**, dicarboxylicacylcarnitines, dopamine, **glucose**, glycerophospholipids, glycolysis, L-aspartate plasmalogen/plasminogen, lysophospholipids, **methylglutarylcarnitine**, **phosphatidylcholines**, **sphingomyelin**, **sphingosine-1-phosphate**, TCA cycle	[80,81,82,83]
**Virus**	Alphainfluenza virus (Influenza)	plasma	H-NMR, GC–MS	**amino acids** and ketone bodies, cAMP, **glucose**, glutathione, lipid, N-acetylglucosamine(O-GlcNAc), purine	[84,85,86,87]
**Virus**	SARS-CoV-2 (COVID19)	plasma, serum	LC–MS	**Bile acids**, **bile acids**, bilirubin, diacylglycerols, **free fatty acid**, **glucose**, glucuronate, glycerol 3-phosphate, **kynurenine**, **lysophosphotidylcholines**, malic acid, monosialodihexosylganglioside, phosphatidylcholines, **sphingomyelin**, triglycerides, **tryptophan**	[14,88,89,90,91,92]

**Table 2:** Metabolomic studies of human vaccine responses. Metabolites induced by more than one vaccine are in bold. TCA, tricarboxylic acid.

Pathogen Type Target	Microbial Target	Vaccine Formulation Studied	Biosample Type	Technique Used	Examples of Metabolites or Metabolic Pathways Perturbed	References
**Bacterium**	*Francisella tularensis*	*F. tularensis* (LVS-DynPort Vaccine)	plasma	LC–MS	2-oxocarboxylic acid, asparagine, **glycolysis, purine**, pyruvate, TCA cycle	[118]
**Bacterium**	*M. tuberculosis*	BCG (Connaught strain)	serum	LC–MS	1,5-anhydroglucitol, alpha-ketobutyrate, de novo **purine synthesis**, glucose processing metabolites, methylguanine, N6-carbomoyltheronyladenosine	[119]
**Virus**	Hantavirus	Hantavax (GreenCross)	serum	LC–MS	16-hydroxyplamitate, arachidonic acid, arginine, benzoate, chenodeoxycholic acid, cholesteryl nitrolinoate, cystathionine, **glutamine** and citrulline, glycine, **histidine**, homocysteine, indole 3-acetaldehyde, isoleucine, leucine, **methionine**, methyl palmitate, N-stearoyl, octanoylcarnitine, phenylalanine, **threonine**, **tryptophan**, tyrosine, ubiquinone-9, valine	[111]
**Virus**	Influenza	Fluzone (2014–2015, 2015–2016) *co-administered with antibiotics	plasma	LC–MS	primary and secondary bile acids, **tryptophan** metabolism	[110]
**Virus**	Varicella zoster (Shingles)	Zostavax	plasma	LC–MS	aldarate, ascorbate, gluconeogenesis, **glycolysis**, inositol phosphate, propanoate, sterol, TCA cycle, **tryptophan**	[11]
**Virus**	Variola virus (Small Pox)	DryVax or ACAM 2000	serum	H-NMR	2-aminobutyrate, alanine, choline, creatinine, fructose, glutamate, **glutamine**, **histidine**, lactate, **threonine**, lysine, **methionine**, propylene glycol, serine	[120]
